# Oral Health for Older Adults: Policy Barriers and Opportunities

**DOI:** 10.1093/geroni/igab046.3420

**Published:** 2021-12-17

**Authors:** Stephanie Sam de Lazaro, Anchee Nitschke Durben, Juliette Kline

**Affiliations:** 1 St. Catherine University, Saint Paul, Minnesota, United States; 2 St. Catherine University, St. Paul, Minnesota, United States

## Abstract

Tooth decay and gum disease are two of the most common chronic health conditions in the United States, are reversible and preventable, and impact approximately 68% of older adults nationwide (CDC, 2021; World Health Organization, 2020). While the Affordable Care Act added provisions to health prevention services, oral health prevention coverage was only included for children, leaving many adults and older adults without coverage (Nasseh & Vujicic, 2017). The research team used a rapid review process using 17 key search term combinations to identify literature in three medical databases (PubMed, CINAHL, and Consumer Health Complete) to identify system and policy level barriers and opportunities to address oral health equity issues for older adults in the United States. 40 articles met inclusion criteria for thematic analysis. Findings revealed three barrier categories: 1) poor oral health literacy of patients and health care providers, 2) reimbursement variability contributing to access and utilization barriers, 3) workforce and scope of practice variability. In addition, four opportunity categories were identified: 1) community-based oral health programming for older adults, 2) new reimbursement models, 3) medical-dental collaborations, and 4) policy and practice act updates. The COVID-19 public health crisis has impacted the implementation of some system and policy level opportunities. However, new health care initiatives specific to Medicare in discussion at the national level provide an opportunity to make some headway on the policy updates needed to address the oral health of older Americans. Findings and implications will be shared with the audience.

